# Improving Operator Depth Vision in Gastrointestinal Endoscopy: A Pilot Study of a New Three‐dimensional Imaging System

**DOI:** 10.1002/deo2.70174

**Published:** 2025-07-16

**Authors:** Xiaoqing Lin, Kohei Ono, Ken Ohata, Etsuko Yamabe, Hidenori Eida, Miyuzen Kanamori, Naoki Tomita, Toshifumi Iida, Susumu Banjoya, Tomoya Kimura, Hiroshi Yamazaki, Koichi Furuta, Nao Takeuchi, Yoshiaki Kimoto, Yuki Kano, Yohei Minato, Shunya Takayanagi, Shinya Nagae, Yohei Ito, Ryoju Negishi, Eiji Sakai, Hideyuki Chiba, Zhen Ding

**Affiliations:** ^1^ Department of Gastrointestinal Endoscopy NTT Medical Center Tokyo Japan; ^2^ Department of Gastroenterology and Hepatology The First Affiliated Hospital Sun Yat‐sen University Guangzhou China; ^3^ Department of Medicine Haukeland University Hospital Bergen Norway; ^4^ Division of Gastroenterology Itabashi Chuo Medical Center Tokyo Japan; ^5^ Yokohama Sakae Kyosai Hospital Federation of National Public Service Personnel Mutual Associations Yokohama Japan; ^6^ Department of Gastroenterology Omori Red Cross Hospital Tokyo Japan

**Keywords:** 3D system, depth perception, distance judgment, gastrointestinal endoscopy, imaging technique

## Abstract

**Background and aims:**

Traditional two‐dimensional (2D) gastrointestinal endoscopy lacks depth perception, leading to potential diagnostic errors. This study evaluates a novel software‐based three‐dimensional (3D) endoscopy system that converts 2D images into 3D, compatible with existing endoscopes.

**Methods:**

A randomized comparative study was conducted with 32 endoscopists at NTT Medical Center. Participants were assigned to perform snaring tasks using either 2D or 3D imaging in short‐ and long‐distance scenarios. Success rates in first attempts were compared between the two groups.

**Results:**

In the long‐distance scenario, the first‐attempt success rate was significantly higher in the 3D group (53.13%) compared to the 2D group (21.88%, *p* = 0.01). The 3D system provided a notable improvement in depth perception and distance judgment, especially for less experienced endoscopists. No significant difference was observed in the time per attempt between the two groups.

**Conclusions:**

The new 3D system enhances depth perception and distance judgment, particularly benefiting less experienced endoscopists.

## 1 Introduction

The invention of the endoscope is one of the greatest advancements in gastrointestinal medicine, enabling direct visualization of the gastrointestinal tract for both diagnosis and treatment. However, the images obtained from traditional endoscopy differ from what the human eye perceives. Depth perception refers to the ability to interpret the three‐dimensional spatial arrangement of objects based on binocular disparity and monocular depth cues. Distance judgment specifically refers to the quantitative estimation of the physical distance between the observer and an object or between two objects. Traditional endoscopy provides two‐dimensional (2D) images and lacks the ability to accurately perceive depth and distance. In contrast, human vision relies on subtle differences between images captured by each eye to create a three‐dimensional (3D) view, enabling depth perception and precise distance judgment. Therefore, beginners need training to develop the ability to “imagine” 3D space from 2D endoscopic images. Misjudgment of space may increase the rate of missed diagnosis and complications, such as perforation or hemostasis failure.

This challenge also exists in laparoscopic surgery. Performing 3D spatial operations under 2D imaging presents unique challenges, making the learning curve for these procedures steeper than that of traditional open surgeries [1, 2]. Operators often experience increased visual and cognitive load due to the loss of depth perception and spatial orientation [3, 4, 5]. To address this, 3D technology has been introduced in laparoscopy, particularly in procedures requiring depth precision and detailed visualization. Numerous studies show that 3D laparoscopy is superior or at least equivalent to 2D in improving operative speed and reducing errors [6, 7], and it benefits trainee education [8]. In video‐assisted thoracic surgery, 3D imaging has been shown to shorten surgical time and reduce intraoperative bleeding [9, 10]. Similarly, in the realm of endoscopy, 3D endoscopy has achieved promising results in procedures such as nasal surgeries, skull base surgeries, and pituitary adenoma resections. Previous research has established that 3D endoscopy surpasses 2D endoscopy in enhancing stereoscopic vision, accurately assessing anatomical structures, and ensuring more precise instrument manipulation [11, 12].

In gastrointestinal endoscopy, 3D imaging is still under exploration. Over the past decade, some trial 3D gastrointestinal endoscopes have been developed and studied. Research suggests that 3D endoscopy enhances early cancer detection [13], improves lesion margin delineation [14], and increases colorectal polyp detection rates [15]. Additionally, ex vivo studies indicate its potential effectiveness and safety in endoscopic submucosal dissection (ESD) [16, 17, 18, 19].

Despite the introduction of 3D technologies in the 1990s, they have not yet been widely adopted in routine clinical practice. Traditional 3D endoscopes rely on dual‐lens and dual‐light systems to capture images for the left and right eyes, making them bulky and difficult to operate. Additionally, due to optical zoom limitations, rigid 3D endoscopes cannot align the images from the left and right eyes horizontally during rotation, preventing the brain from merging images correctly. Previous studies also reported that traditional 3D endoscopes can cause eyestrain and dizziness [19, 20, 21]. Furthermore, these systems lack compatibility with different endoscope functions, diameters, and angles, restricting their clinical applications. The combination of limited functional compatibility, operator discomfort, and relatively high cost [2, 22] has prevented 3D gastrointestinal endoscopes from achieving commercial viability to date.

To overcome these barriers, a new software‐based 3D system has been developed using the “keep 2D, see 3D” concept. It generates 3D images from conventional 2D endoscopic footage by extracting depth information and creating depth maps. Its key advantage is compatibility with existing platforms, allowing seamless switching between 2D and 3D views with standard 3D glasses. More comparisons between the two systems have been published in previous articles [23].

To evaluate the effectiveness of this new 3D endoscope system, we designed an ex vivo comparative study to investigate the depth perception and distance judgment between 2D and 3D imaging.

## 2 Methods

### 2.1 Participant

This is a randomized comparative study. All endoscopists at NTT Medical Center participated in this study, including overseas trainees. Experienced endoscopists were defined as those who had independently performed > 2000 colonoscopies; intermediate endoscopists were defined as those who had independently performed 1000–2000 colonoscopies; novice endoscopists were defined as those who had independently performed <1000 colonoscopies.

### 2.2 Equipment

**FIGURE 1:**
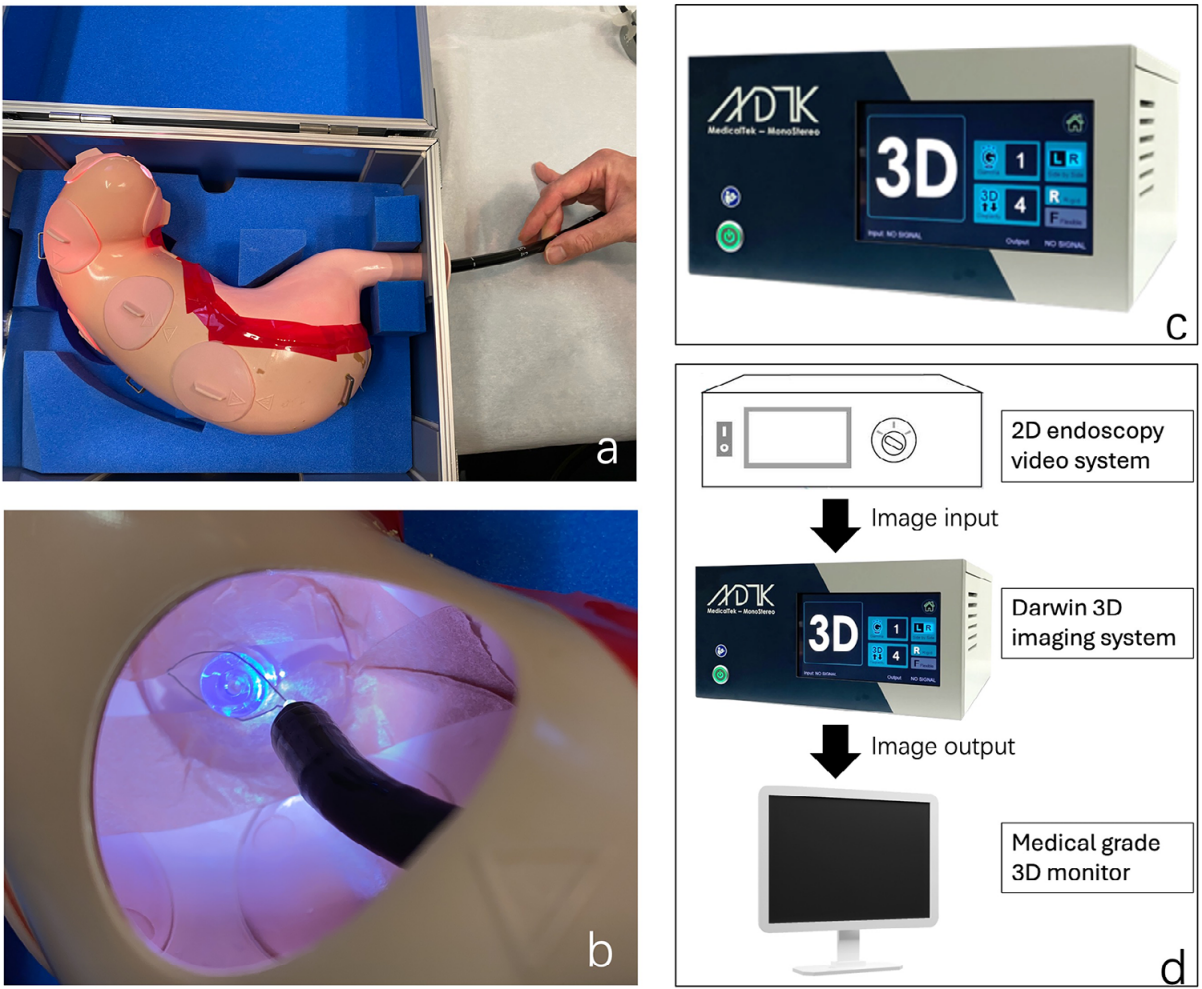
Scenario of the experiment and three‐dimensional (3D) imaging system used in this study. (a) Experiments in this study were conducted using a simulated stomach model for training purposes. (b) A bottom was installed in the stomach model; the task for the operator was to use a snare to catch the bottom. (c) Darwin 3D imaging system. (d) Schematic diagram illustrating the connection between the endoscope and Darwin.

**FIGURE 2:**
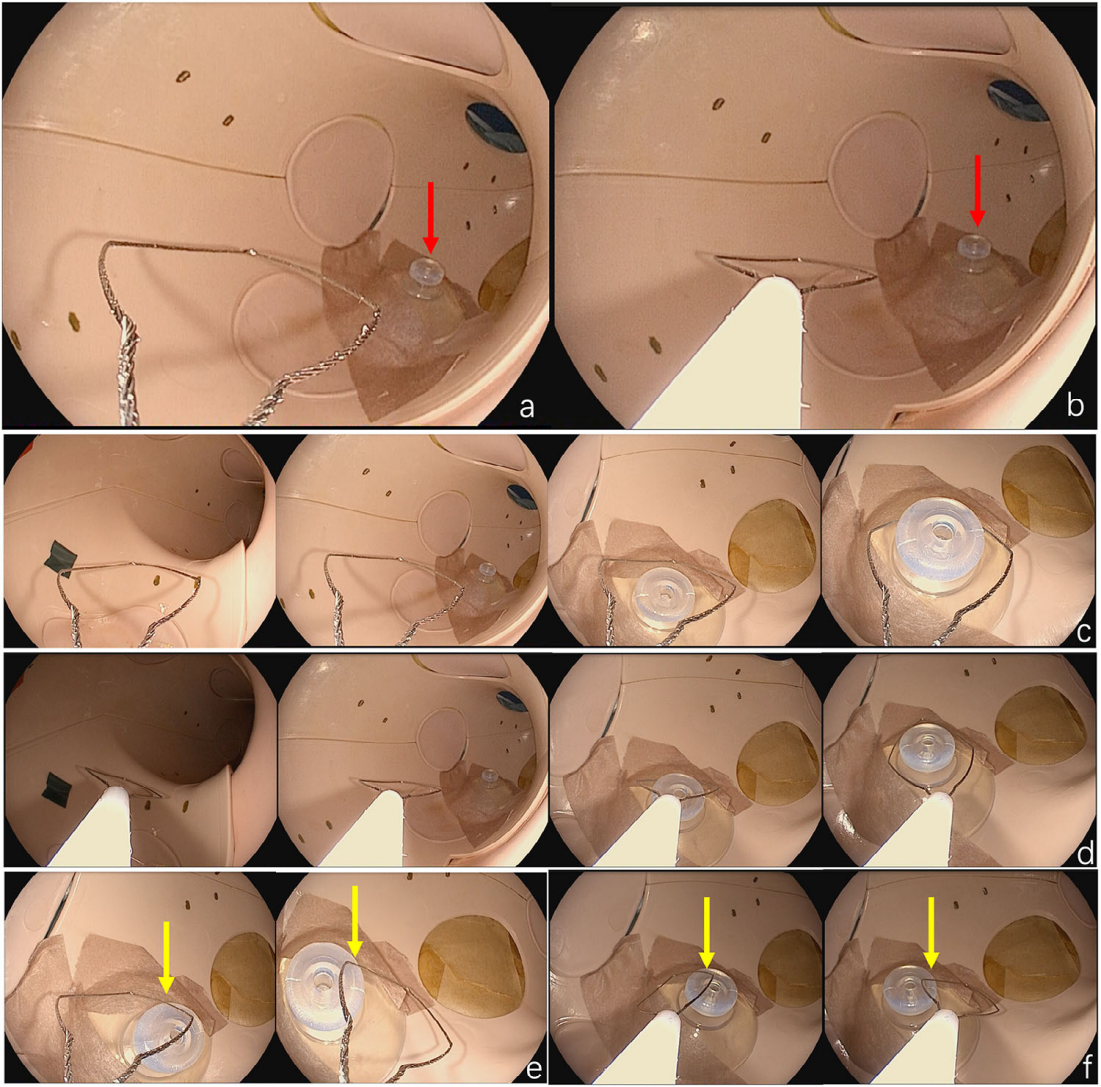
Examples of snare setup and successful/failed scenarios. (a) Setup of the “short‐distance” configuration. The protrusion length of the snare's sheath is fixed at 0 cm. The red arrow indicates the target of the snare. (b) Setup of the “long‐distance” configuration. The protrusion length of the snare's sheath is fixed at 3 cm. The red arrow indicates the target of the snare. (c) Schematic diagram of the experiment in a short‐distance scenario. Start from the “cardia” of the stomach model—move to the target—close to the target—successfully catch the target without touching the surface of the target. (d) Schematic diagram of the experiment in a long‐distance scenario. Start from the “cardia” of the stomach model—move to the target—close to the target—successfully catch the target without touching the surface of the target. (e) Schematic diagram of operation “failure” at short distance. The yellow arrow indicates where the snare “touch” the target surface. (f) Schematic diagram of operation “failure” at a long distance. The yellow arrow indicates where the snare “touch” the target surface.

As shown in the figure (Figure 1a,b), the target was installed in a stomach model. The protrusion sheath length of the snare was fixed at either 0 cm for the short‐distance scenario or 3 cm for the long‐distance scenario (Figure 2a,b). The task for the participants was accurately snaring the target at different distances. To accurately reflect participants' judgment of distance, the experiment required participants to snare the target from directly above, without touching the surface of the target during the process (Figure 2c,d). If the snare touched the surface, it was considered cheating and recorded as a failure (Figure 2e,f).

The 3D imaging system we used in this study was a Darwin 3D endoscope from the MedicalTek company. By using optical and computer vision methods to generate 3D vision, the single light from the endoscopy system creates a Lambert light distribution [24] on the projection plane and captures the light image from the endoscope. Objects at different distances result in different light distributions. The device calculates the shading, light, and motion information from continuously captured images to generate depth values. The high‐definition image of the 2D endoscopy combines with the depth map to generate different disparity views as left and right camera images. The system connects with conventional 2D endoscopy systems and utilizes graphics processing unit computing to achieve real‐time 3D conversion.

The endoscopy system and endoscope used in the experiment were: PENTAX SMC‐V13, *PENTAX Medical (Japan)*; EG29‐i10, *PENTAX Medical (Japan)*.

The 3D system used was: Darwin 3D endoscope, *MedicalTek Co., Ltd*. (Figure 1c,d).

The snare used in the experiment was: Polypectomy Snare, Product No. AG6.507AO.0652.000.D, *AGS MEDTECH* (China).

### 2.3 Study Design

All participants were randomly assigned to either the 2D‐preceding group or the 3D‐preceding group. The grouping determined whether participants would perform the 2D or the 3D operation first. A research coordinator (PLT Co., Ltd.), independent of the procedure, randomly assigned participants in a one‐to‐one ratio to either the 2D‐preceding group or the 3D‐preceding group. Randomization followed a minimization method with stratification based on the operator's experience level (experienced or less‐experienced). Allocation remained concealed from the operators until the moment before the procedure. The coordinator disclosed the assignment to the endoscopists immediately prior to the procedure.

**FIGURE 3:**
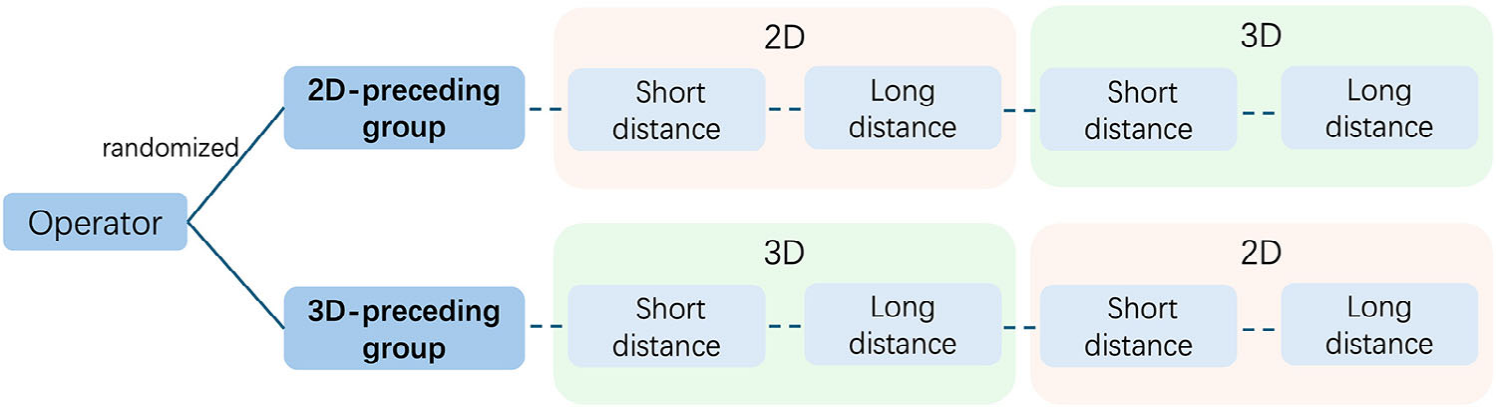
Study flowchart. Operators were randomized into groups according to this process. Based on the groupings, they completed the experiments in different sequences under two‐dimensional (2D) and three‐dimensional (3D) scenarios.

As shown in Figure 3, after randomization, participants performed both short‐distance and long‐distance operations under 2D and 3D endoscopy. Starting from the cardia, each attempt was counted from the start to the successful capture of the target or failure of the operation. During the whole process of the study, there was only one “referee” to judge the success or failure of the operation. When any part of the snare touched the surface of the target before it was caught, we judged it as a failure (Figure 2e,f). Each setting's endpoint was either achieving success or reaching a maximum of 5 attempts. The operation time for each attempt was recorded. Before starting the experiment, all operators were allowed to practice once and adjust the distance between themselves and the 3D screen to obtain a clear 3D image.

The primary outcome of this study was to compare the success rate of the first attempt between the 2D group and the 3D group. The secondary outcome was to compare the success rate within five attempts between the two groups. *IBM SPSS Statistics 27.0* was used for analysis. Chi‐square test was used to compare success rates, and a paired t‐test was used to compare operation times between the 2D and 3D groups. A *p*‐value < 0.05 was defined as statistically significant.

## 3 Results

A total of 32 endoscopists participated in this study, including 18 males and 14 females. After randomization, the 2D‐preceding and the 3D‐preceding groups had 16 participants each. The distribution of experienced endoscopists, intermediate endoscopists, and novice endoscopists was 16:8:4.

The number of experienced, intermediate endoscopists and novice endoscopists was 6, 6, and 4, respectively, in the 3D‐preceding group, and 10, 6, and 0 in the 2D‐preceding group. There was no statistical difference between the two groups (*p* = 0.082). The male‐to‐female ratio was identical in both the 2D and 3D preceding groups, at 7:9.

### 3.1 Success Rate

As shown in Table 1, in the short‐distance scenario, the success rate of the first attempt for the 2D and 3D visualization groups was 21.88% (7/32) vs. 43.75% (14/32), *p* = 0.06. In the long‐distance scenario, the success rate of the first attempt for the 2D and 3D visualization groups was 21.88% (7/32) vs. 53.13% (17/32), *p* = 0.01.

**TABLE 1:** Comparison of success rate between the two‐dimensional (2D) visualization group and the three‐dimensional (3D) visualization group.

		2D visualization％, (95%CI)	3D visualization％, (95%CI)	χ^2^	*p*‐Value
**Success rate in the first attempt**	Short‐distance	21.9% (7/32) (10.7%, 39.0%)	43.8% (14/32) (28.1%, 60.7%)	3.473	0.06
	Long‐distance	21.9% (7/32) (10.7%, 39.0%)	53.1% (17/32) (36.4%, 69.1%)	6.667	0.01^*^
**Success rate within five attempts**	Short‐distance	81.3% (26/32) (64.3%, 91.5%)	84.4% (27/32) (67.8%, 93.6%)	0.110	0.74
	Long‐distance	90.6% (29/32) (75.0%,97.5%)	93.8% (30/32) (78.8%‐99.3%)	0.217	0.64

* *p* < 0.05

For the success rate within five attempts, there was no difference in both the short‐distance scenario and the long‐distance scenario (*p*‐values were 0.74 and 0.64, respectively).

### 3.2 Subgroup Analysis

As shown in Table 2, in the experienced endoscopist group, the success rate in the 3D visualization group was slightly higher than that in the 2D visualization group for both short‐distance and long‐distance operations, but there was no statistically significant difference. Among novice operators, the 3D visualization group demonstrated a significantly higher first‐attempt success rate than the 2D visualization group in long‐distance scenarios (*p* = 0.03). For intermediate operators, the 3D group also had a higher first‐attempt success rate in short‐distance scenarios (*p* = 0.01), while in long‐distance scenarios, there was no statistically significant difference between the two groups (*p* = 0.08). Among experienced operators, there was no significant difference in success rates between the 3D and 2D visualization groups in either scenario.

**TABLE 2:** Subgroup analysis for experienced/intermediate/novice group.

		2D visualization％, (95%CI)	3D visualization％, (95%CI)	χ^2^	*p*‐Value
**Novice endoscopists**	Short‐distance	25.0% (1/4) (3.4%, 71.1%)	0 (0/4) (‐5.6%, 54.6%)	1.129	0.29
	Long‐distance	0% (0/4) (‐5.6%, 54.6%)	75.0% (3/4) (27.0%, 99.9%)	4.8	0.03^*^
**Intermediate endoscopists**	Short‐distance	0% (0/12) (‐0.67%, 37.5%)	41.7% (5/12) (17.7%, 68.3%)	6.32	0.01^*^
	Long‐distance	16.7% (2/12) (3.5%, 46.0%)	50.0% (6/12) (25.4%, 74.5%)	3	0.08
**Experienced endoscopists**	Short‐distance	37.5% (6/16) (18.5%, 61.4%)	56.3% (9/16) (33.1%, 76.9%)	1.129	0.29
	Long‐distance	31.3% (5/16) (13.9%, 55.9%)	50.0% (8/16) (28.0%, 72.0%)	1.166	0.28

* *p* < 0.05

For the success rate within five attempts, there was no difference in both the short‐distance and long‐distance scenarios, in both the experienced and less‐experienced endoscopist groups.

### 3.3 Difference in Success Rate Between 2D‐preceding and 3D‐preceding Groups

A comparison was made between the success rates of the 2D‐preceding and 3D‐preceding groups under each setting. The results showed no statistically significant difference in the first‐attempt success rate between the 2D‐preceding and 3D‐preceding groups under each setting (p > 0.05), as shown in Table 3.

**TABLE 3:** Success rate in first attempt between the two‐dimensional (2D)‐preceding group and the three‐dimensional (3D)‐preceding group.

	2D‐preceding％, (95%CI)	3D‐preceding％, (95%CI)	χ^2^	*p*
**Short‐distance 2D**	18.8% (3/16) (5.8%, 43.8%)	25.0% (4/16) (9.7%, 50.0%)	0.183	0.69
**Short‐distance 3D**	43.8% (7/16) (23.1%, 66.9%)	43.8% (7/16) (23.1%, 66.9%)	0.000	1.00
**Long‐distance 2D**	12.5% (2/16) (2.2%, 37.3%)	31.3% (5/16) (13.9%, 55.9%)	1.646	0.20
**Long‐distance 3D**	50.0% (8/16) (28.0%, 72.0%)	56.3% (9/16) (33.2%, 76.9%)	0.125	0.72

### 3.4 Time Duration

In the short‐distance scenario, the average time duration of each attempt in the 2D visualization group was 19.04 ± 7.26 s, while in the 3D visualization group it was 20.07 ± 9.92 s, *p* = 0.55. In the long‐distance scenario, the average time duration of each attempt in the 2D visualization group was 19.03 ± 11.69 s, 3D visualization group 19.69 ± 10.74 s, *p* = 0.67.

## 4 Discussion

The theoretical basis of our experimental design is that during endoscopic procedures, operators with accurate distance judgment can precisely grasp the target with fewer attempts, while those with less accuracy require multiple tries. Judgment of three‐dimensional space not only affects operation time but can also impact the safety of the procedure. For example, in ESD surgery, repeated inaccurate attempts to stop bleeding can cause perforation or delayed perforation due to increased coagulation attempts. In this study, the first‐attempt success rate most accurately measures the operator's distance judgment accuracy. Data analysis shows that compared to the 2D group, the 3D group has a significantly increased success rate at long distances, indicating that 3D imaging may provide better depth perception and distance judgment than 2D imaging at long distances.

Since each operator performed both 2D and 3D operations, prior experience could potentially make subsequent operations easier. To minimize this bias, we randomized the grouping of the operators. To verify whether 2D‐preceding or 3D‐preceding affected the operational outcomes, we compared the first‐attempt success rates of the 2D‐preceding and 3D‐preceding groups in each experimental setting. The results demonstrated that the data between the two groups were comparable, indicating that the superiority of the 3D imaging system over the 2D imaging system in this study is reliable. Junior endoscopists have less experience in imagining 3D space using conventional endoscopes due to a lack of experience. This disadvantage is more pronounced in clinical scenarios that require high spatial judgment ability. Our study results indicate that while there was no difference in trial success rates between the 2D and 3D groups for experienced endoscopists; however, there is a noticeable difference in trial success rates between the 2D and 3D groups for less‐experienced endoscopists, especially in long‐distance trials. Since long‐distance operations require higher spatial judgment ability from the operator, the trial results confirm that 3D imaging can compensate for the spatial judgment deficiency of junior endoscopists. Therefore, the application of a 3D imaging system may be more beneficial to less‐experienced endoscopists.

To clarify whether the operators deliberately slowed down the operation speed to achieve a better success rate during 3D operations, we compared the operation times of each setting in the 2D and 3D groups. The results showed that the average operation time per attempt was comparable between the two groups. This indicates that the operators did not deliberately perform more carefully during 3D operations, and the success rate data in this study are reliable.

The main advantage of this study is that it is the first to demonstrate the effectiveness of this new 3D system in improving depth perception. This study was conducted in a simulated gastric model using clinically realistic instruments and included both short and long‐distance operations to simulate different clinical scenarios as much as possible. The design of “grasping the target from directly above” reflects the operator's distance judgment ability and greatly reduces “cheating” behavior by using surrounding references. This study used random grouping to minimize the positive impact of operational experience on subsequent operations, making the results reliable.

However, this study has some limitations. First, we did not conduct a formal pre‐study power calculation; the sample size was determined based on practical considerations. A formal power analysis would strengthen future work. Secondly, due to the differences between 2D and 3D endoscopy, a double‐blind trial was not possible. Therefore, potential measurement errors (i.e., bias towards the 3D view) may occur during the study. Although our comparison showed that the operation times of the 2D and 3D groups were comparable, suggesting similar carefulness when using different endoscopes, this does not completely rule out the possibility of measurement errors. Thirdly, this is a single‐center study. Despite including physicians with different experiences from different countries, the small sample size made it unclear whether our results could be generalized to all endoscopists. Future studies with larger sample sizes are needed to confirm our results. Fourthly, we chose to define “long distance” as 3 cm for several reasons. In most endoscopic procedures, the lens is positioned close to the target, with longer distances required only in specific scenarios, such as biopsies of gastric fundus lesions or ESD of the lesser curvature of the gastric body. In these cases, 3 cm is an approximate working distance. Furthermore, during the early experimental design stage, we observed a high failure rate for both 2D and 3D visualization at longer distances, which limited our ability to compare their differences due to a floor effect. But we believe that examining a broader range of distances could provide a more comprehensive understanding of 3D imaging effectiveness. Future studies exploring the performance of 3D imaging across varying and greater distances are needed. Finally, this study was conducted in a simulation, which differs from the real clinical environment. Future clinical studies are needed to further verify the effectiveness of the 3D imaging system in clinical practice, along with comprehensive cost‐effectiveness analyses.

In summary, the new 3D imaging system can improve the operator's depth perception, and this advantage is more pronounced in long‐distance operations. Additionally, the application of the new 3D imaging system may be more beneficial to junior endoscopists.

## Ethics Statement

Approval of the research protocol by an Institutional Review Board: N/A.

## Consent

N/A.

## Conflicts of Interest

The authors declare no conflicts of interest.

## Clinical Trial Registration

N/A.
